# A Localized Phage-Based Antimicrobial System: Effect of Alginate on Phage Desorption from β-TCP Ceramic Bone Substitutes

**DOI:** 10.3390/antibiotics9090560

**Published:** 2020-08-31

**Authors:** Rached Ismail, Natalia D. Dorighello Carareto, Jean-Christophe Hornez, Franck Bouchart

**Affiliations:** LMCPA—Laboratoire des Matériaux Céramiques et Procédés Associés, EA 2443, Université Polytechnique Hauts-de-France, F-59313 Valenciennes, France; rached.ismail@uphf.fr (R.I.); natalia.dorighellocarareto@uphf.fr (N.D.D.C.); jean-christophe.hornez@uphf.fr (J.-C.H.)

**Keywords:** biomaterials, bacteriophage, phage therapy, beta-tricalcium phosphate, alginate

## Abstract

Tricalcium phosphate (TCP) is a prosthetic material commonly used as a bone substitute to repair osteoarticular diseases and injuries. In this type of bone reconstruction surgery, antibiotics remain the common preventive and therapeutic treatment for bacterial infection. Nevertheless, the emergence of multi-resistant strains requires complimentary or alternative treatments. Today, one of the promising alternative approaches is phage therapy. Phages are bacterial viruses that have several advantages over chemotherapy, such as the specificity of bacterial strain, the absence of side effects, and a rapid response. In this work, we studied the impact of alginate hydrogels for overlaying λvir-phage-loaded β-TCP ceramic bone substitutes, delaying the phage desorption. The results show that the use of a 1% alginate–CaCl_2_ hydrogel overlapping the β-TCP ceramic pellets leads to higher initial phage concentration on the material and extends the released time of phages to two weeks when compared with control pellets. These alginate-coated biomaterials also generate faster bacterial lysis kinetics and could therefore be a good practical prosthetic device for bone and joint surgeries by allowing local treatment of bacterial infections with phage therapy for a longer period of time.

## 1. Introduction

Phages are viruses that specifically infect bacteria. They were discovered in the early 20th century [1,2] and have been used to treat and prevent bacterial infections since then [3,4]. However, after the discovery of penicillin, the use of antibiotic drugs became widespread due to their high efficacity over a wide spectrum and their low production cost [5]. At that point, except in some Eastern European countries, phage therapy was unheeded as the antibiotic age was beginning [6,7]. With the increase of high incidence of multidrug-resistant (MDR) bacteria, we may approach what could be a post-antibiotic era in medicine [8]. Consequently, the properties and therapeutic potential of phages have been rediscovered [9,10] and have recently become an active area of interest as they have shown great promises as alternative treatments or in combination with antibiotic therapy [11]. However, the lack of investment and testing in the use of phages as therapeutic agents means that many questions remain open regarding their potential as a method of microbial control and antimicrobial treatment. Currently, the high specificity of phages can be cited as one of the most important advantages over antibiotics and, unlike antibiotics, they have low inherent toxicity to humans [12,13]. Recently, several studies have even demonstrated the presence and the protective role of “endogenous” phages in mammalian organisms [14,15] confirming the hypothesis of a protective action of phages against bacterial infections not only in the human gut, but also lymph and blood [16].

Normally, antibiotics and, in some cases, phages are administered orally [17], intravenously, or intraperitoneally as part of preventive or therapeutic antimicrobial treatment in animals and humans [18]. Microencapsulation of phages has been proposed [19], especially for oral administration, because during the passage of phages through the gastrointestinal tract, they are exposed to a complex chemical and biological environment that could affect their therapeutic efficacy [20]. Nevertheless, in the case of bone graft, the engineering of implants to prevent local infection is a current topic of study [11]. Essentially, 2–5% of all procedures involving implants are followed by bacterial infection, and in the case of open fractures this percentage can be as high as 30% [18]. Meurice et al. [21] proposed the use of bacteriophage-impregnated bone cements as an alternative to antibiotic prophylactic treatments. They showed that λvir phage, which is a specific phage for *Escherichia coli*, has a good interaction in vitro with hydroxyapatite (HA) and beta-tricalcium phosphate (β-TCP) ceramics pellets. HA and β-TCP have structural features and chemical compositions close to that of the mineral in bone that allows their use as bone substitutes in orthopedic and dental surgery. As prophylactic treatments, it is important to ensure a high phage retention in the ceramic pellets after a bone transplantation. This strategy allows the direct introduction of phages on the implantation site. From the first hours of use, a large concentration of phages is released from ceramic biomaterials. It is therefore important to improve the control of their release over time to ensure prolonged control of a potential local infection. For this purpose, a solution is proposed to coat the phages into an encapsulating hydrogel.

Alginate is considered to be a common system of encapsulation [22]. Alginate is a natural polysaccharide, a block co-polymer composed by α-ʟ-guluronic acid and β-D-mannuronic acid residues, linearly linked together [23]. It can be produced from sources of algae or bacteria. Commercially available alginates are mainly derived from different species of brown algae [22]. It can be isolated from *Pseudomonas* or *Azotobacter*, although bacteria alginates are generally not economically viable for commercial applications [24]. When multivalent cations (e.g., Ca^2+^ and Ba^2+^) are added to an aqueous alginate solution, they bind adjacent alginate chains, forming ionic interchain bridges that cause a fast sol–gel transition [25]. This ability has allowed alginate to act as natural agent for cell microencapsulation [26]. Moreover, Barros et al. demonstrated in a recent publication [27] that hydrogels formed by alginate combined to nano-hydroxyapatite enhance osteogenic activation and could maximize bone tissue regeneration. In this work, we propose the use of alginate–CaCl_2_ hydrogel as a coating to the λvir-phage-loaded β-TCP ceramics in order to enhance phage retention on the material and hence allow a more controlled (sustainable) and longer release of the phages.

## 2. Results

### 2.1. SEM Observations of the Hydrogel on Alginate Covered Pellets

**Figure 1:**
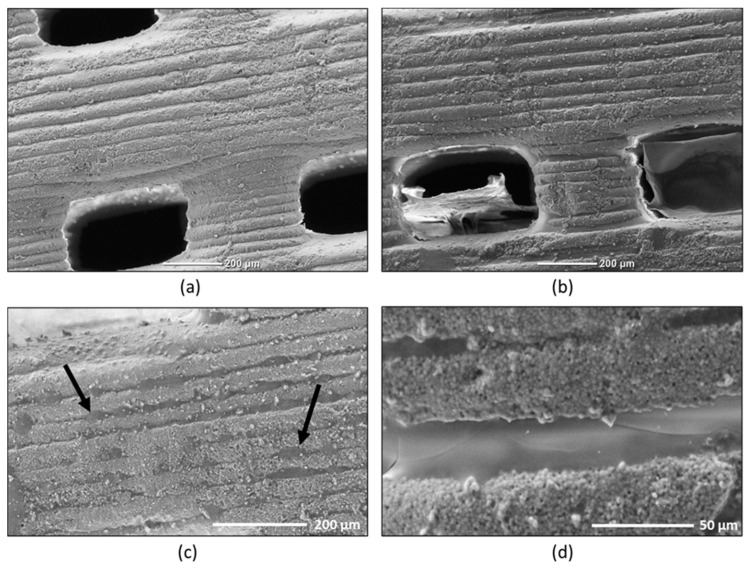
SEM observations of the surface of the alginate-covered pellets. (**a**) Overview micrograph of the control pellets; (**b**–**d**) micrographs at different scales of the 1% alginate–CaCl_2_-coated pellets.

SEM analyses were used to verify the surface structure of the pellets. Figure 1a,b present an overview micrograph of the control and 1% alginate–CaCl_2_-coated pellets. The absence and presence of alginate is well represented in both Figure 1a,b, respectively, in the regions where the macropores are visible. One can note in Figure 1b the shrunken alginate + CaCl_2_ hydrogel. In fact, this shape appears as the result of desiccation from the preparation of the samples to be observed under the electron microscope.

The micrographs in Figure 1a–d reveal the presence of layers arranged in evenly-spaced bands on the pellet surface. Undeniably, the linear movement of the stereolithography apparatus (SLA) printer superimposed the layers of TCP slurry to create the pellet scaffold.

Figure 1b–d show the micrographs of a pellet coated with a 1% alginate–CaCl_2_ hydrogel. The alginate–CaCl_2_ hydrogel is an ionically-cross-linked one. Alginate that is negatively charged combines with the Ca^+2^ forming an insoluble complex [25]. Indeed, the presence of this complex on the pellet surface is well represented, as shown in Figure 1c by the black arrows. Certainly, the alginate–CaCl_2_ hydrogel covered the entire pellet surface, but, as explained before, the vacuum created in the vacuum chamber, used to deposit the Ag on the pellet surfaces, undeniably changed the shape of the alginate–CaCl_2_ coating. In the SEM images, this was visible only in the hollow formed due to the printing technique.

### 2.2. Phage Loading of the Inoculated Ceramic Pellets

The number of phages loaded on the β-TCP pellets was determined as described in Section 4.1.2. The pellets were then tested for lytic activity in triplicate to determine the effectiveness of this method and to demonstrate the total release of the phages from the β-TCP materials. The prepared alginate/phage solutions did not interfere with the repeatability of the amount of phage loaded on the ceramic pellets, nor the reproducibility, as the results are consistent with previous results obtained by Meurice et al. [21] and Bouchart et al. [28]. The method used to introduce phages into the pellets demonstrated significant reproducibility. The absence of residual phages in the pellets was confirmed by OD_269_ measurements. A contact test with a two-generation liquid culture of *E. coli* was also performed. The titration of the phages present in the pellets is therefore significant and reproducible.

The quantity of phages loaded on the pellets (Table 1) was significant assuming the initial phage concentration (1 × 10^10^ PFU/mL).

As expected, the alginate–CaCl_2_ hydrogel showed a positive influence on the initial phage concentration in the pellets. In fact, the 1% alginate–CaCl_2_ hydrogel allowed a phage concentration 1 log higher than on the uncoated control pellets. Compared to the initial concentration observed on the pellets by Meurice et al. [21], it was significantly higher, demonstrating an important retention action of the alginate–CaCl_2_ hydrogel of the phages into the ceramic pellets.

### 2.3. Influence of the Percentage of Alginate on the Phage Retention on the Pellets

**Figure 2:**
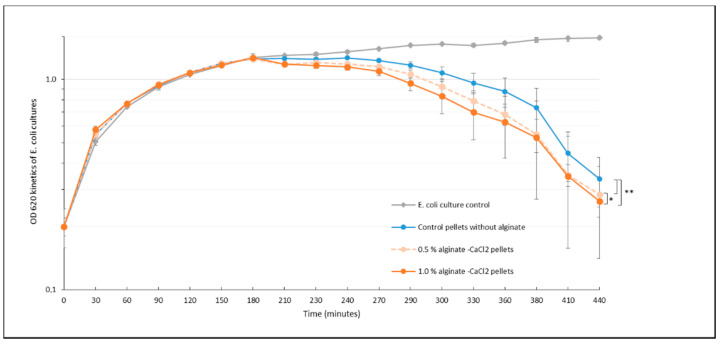
Lytic kinetics of bacterial cultures in the presence of phage-loaded β-TCP pellets coated with different alginate concentrations after 3 h of washing in LB (37 °C, 170 rpm) (*n* = 3 × 2). *, Significantly-similar groups of results and **, significantly-different groups of results with *p*-value < 0.05. The grey kinetic corresponds to the results for the control culture of *E. coli*.

Different percentages of alginate/phage solutions were prepared to assess the effect of alginate concentration on phage retention into the pellets. These percentages were selected in order to obtain malleable overlaying of alginate hydrogels all around the β-TCP pellets without encapsulating effects. The results, shown in Figure 2, demonstrated that at *t* = 210 min, the alginate–CaCl_2_-hydrogel-coated pellets began to generate bacterial lytic kinetics around an hour before the alginate-free control pellets.

From a quick rinsing to a 6-h wash, the β-TCP pellets coated with alginate–CaCl_2_ exhibited a higher lytic activity than the control pellets. Moreover, the decrease in lytic activity observed after 3 h of washing seemed to be moderated by the presence of the hydrogel. This slowdown was also observed with higher concentrations of alginate (data not shown) but did not lead to significantly-different efficiencies from those of 1% alginate hydrogels. At *t* = 24 h, all the lytic kinetics reached the total lysis point around OD_620_ = 0.2.

### 2.4. Impact of Alginate on the Phage Retention within Two Weeks

**Figure 3:**
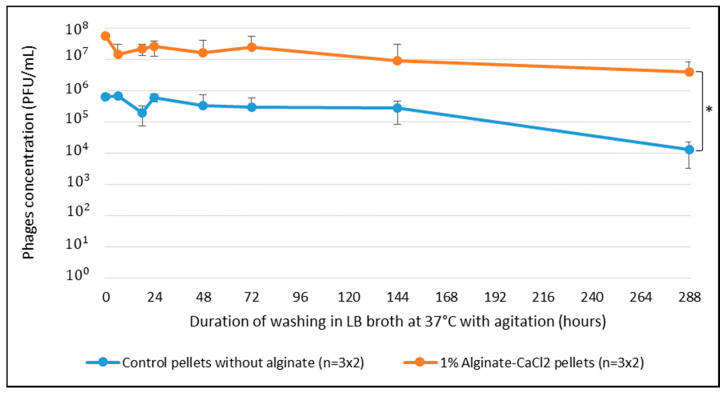
Evolution of phage concentrations in control and 1%-loaded CaCl_2_–alginate-hydrogel-coated β-TCP pellets after LB broth washing at 37 °C, under 170 rpm agitation for two weeks. *, Significantly-different groups of results with *p*-value < 0.05.

The impact of the alginate–CaCl_2_ hydrogel retention on the phage load of the β-TCP pellets after a period of time under mimicked in vivo conditions (37 °C, light flow of nutritive broth) was assessed for two weeks. The results are displayed on Figure 3 and demonstrate the retention action of the alginate–CaCl_2_ hydrogel coating on the phage concentration loaded in the pellets as a function of wash time.

The retention efficiency observed after 6 h washing of the alginate covered phage-loaded β-TCP pellets was confirmed and was maintained for two weeks. The capability of the hydrogel to maintain a higher lytic activity of the phage-loaded biomaterials towards bacteria planktonic cultures appears to be sustainable in the long run. Although the alginate-free control pellets showed a critical difference from the beginning, the lytic activity remains for 1 week of washing but tends to decrease more significantly after this time as shown on Figure 3.

These results also tend to show that the higher level of lytic activity of the alginate pellets could be explained by the retention effect of hydrogel on phages.

## 3. Discussion

Λvir-phage-loaded β-TCP pellets had already demonstrated lytic activity on *Escherichia coli* liquid cultures in previous work. In fact, Meurice et al. [21] previously confirmed the lytic activity with a precision on the phage loading concentration on the ceramic pellets. The present study focused on assessing λvir-phage desorption from the pellets. The results showed a decrease of 2-log between initial phage load and load titrated after 2 weeks for control pellets without alginate–CaCl_2_ hydrogels. This inherent desorption could lead to a decrease of the efficiency and lytic response of the λvir-phage-loaded β-TCP pellets in the case of long prophylactic treatments. This work also demonstrated the relevance of the use of a hydrogel as a protective encapsulation of the λvir-phage-loaded β-TCP pellets to control the natural phage property of desorption from the calcium phosphate bioceramics. Indeed, even if there is still a reduction in the initial phage load, it is reduced to 1 log after 2 weeks for the 1% alginate–CaCl_2_-hydrogel-coated pellets. Reducing the desorption of the phages loaded on biomaterials could lead to a longer period of pre- and postoperative treatments and thus, decreasing risks of infections.

These first results show a promising impact of alginate as a protective hydrogel around the phage-loaded bioceramics. Alginate-covered λvir-phage-loaded β-TCP pellets not only showed a higher lytic activity on *E. coli* throughout the two weeks but also demonstrated a better retention of the phages on the pellets, thereby limiting the dispersion of the phages within the environment of the pellets. A higher initial phage concentration in the pellets also meant a higher lytic activity towards the targeted bacteria at the first moment of contact. This immediate antimicrobial response was also demonstrated by Bouchart et al. [28] against *Staphylococcus aureus* biofilms.

These results could be explained mechanically by a protective, though permeable, coating covering the λvir phages on the surface of the 3D ceramic patterns. As shown by microscopy (Figure 2), the layers formed by the alginate hydrogel on the asperities of the pellets could explain the protective action of the hydrogel on the phage load. This property could be interesting when the alginate-covered biomaterials are placed in a fluidic environment, for example in vivo to maintain the lytic activity of the bone substitute and for the safety of osteoblast eukaryotic cells, as suggested by Bouchart et al. [28]. Moreover, the use of alginate did not affect the lytic activity of the phage and led to better lytic efficiency of our biomaterials from the start. The use of this hydrogel as an encapsulation of the bioceramics should be an interesting prospect to prevent bacterial colonization of the material and biofilm formation on the prosthesis by enhancing the duration of the biomaterial efficacity. In the long term, the biodegradability of the alginate could also allow a protection of the prosthesis and its immediate environment by gradually releasing the phages. Though encapsulation has already been studied, the incorporation of phages in a biodegradable matrix is still an exploratory work to be pursued. Currently, several recent studies show interesting results with hydrogels only as bone cements. However, exclusive hydrogel use for biomedical purposes leads to technological limitations as media and pH have a great influence on the gelling and stability of hydrogels [29]. Alginate–ceramic composite hydrogels are an interesting prospect as a delivery system [27] but for the lytic activity to endure over time, another approach with solid material structures should be adopted. Alginate films and hydrogel coating on ceramic biomaterials appears to be a promising way to provide phage-based antimicrobial activities. Moreover, the action of phages loaded inside these alginate-combined biomaterials could be enhanced by providing different phages in a cocktail [28] or by combining them synergistically with antibiotics [30] and other chemically-active antimicrobial molecules [31].

Allowing local and immediate antimicrobial activity against bacterial infections or offering long-term protection against known potential contaminations and biofilms colonization, alginate–CaCl_2_-coated bioceramics are innovative and versatile tools that could lead to therapeutic prostheses against postoperative joint and bones infections.

## 4. Materials and Methods

### 4.1. Bacteria Growth Conditions and Phage-Killing Titer Preparation

#### 4.1.1. Bacteria Strain, Media, and Growth Conditions

The bacterial strain used in this study was a λvir-phage-sensitive K12 strain of *Escherichia coli* (A324) [32]. Liquid cultures were grown overnight at 37 °C under 170 rpm agitation, in lysogenic broth (LB) (bactotrypton 5 g/L, yeast extract 10 g/l, NaCl 5 g/L, pH 7.2). For enumeration and solid tests, bacteria were grown on LB Agar (LB and agar 15 g/L) at 37 °C for 24 h.

#### 4.1.2. λvir-Phage Stock and Titration

The λvir-phage titer was prepared by infection of *Escherichia coli* K12 strain (A324) liquid culture and stocked at 4 °C. The titration was performed by counting bacterial lytic plaques on solid assays with doubled-layered LB/R-Top agar plates, this low percentage agar solid medium promoting λvir-phage diffusion. Next, 100 µL of each λvir-phage stock dilution was mixed with 100 µL of *E. coli* A324 liquid culture at 37 °C for 20 min and then added to 3 mL of R-Top medium (bactotrypton 10 g/L, yeast extract 1 g/L, NaCl 8 g/L and agar 8 g/L) and poured on an LB agar plate [33]. The lysis plaques were counted at 37 °C after 24 h to estimate the PFU (plaque forming unit)/mL concentration of phages in the stock.

### 4.2. β-TCP Pellet Preparation

#### 4.2.1. Synthesis and Stereolithographic Shaping of the β-TCP Pellets

**Figure 4:**
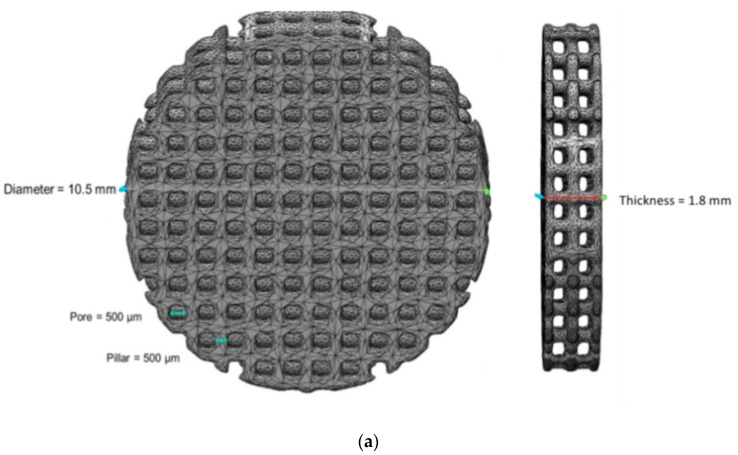

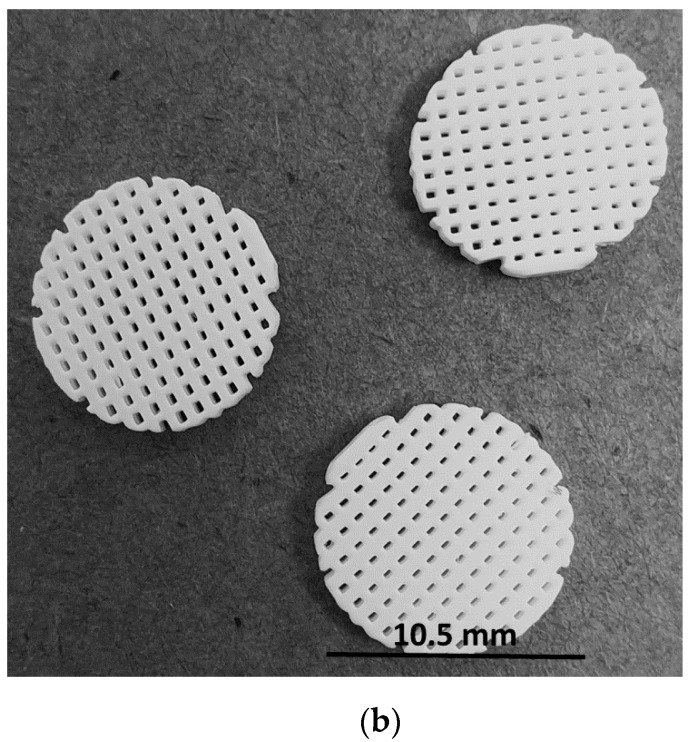
3D printed macroporous β-TCP pellets used in this study. (**a**) 3D design of the pellets and (**b**) the printed pellets.

Synthetic β-Ca_3_(PO_4_)_2_ (β-TCP) powder was prepared by aqueous precipitation using di-ammonium phosphate solution (NH_4_)_2_HPO_4_ (Carlos Erba, Val de Reuil, France) and calcium nitrate solution Ca(NO_3_)_2_·4H_2_O (Brenntag, Tournan en Brie, France) at 35 °C and pH 6.4 with an initial Ca/P molar ratio of 1.5 and an aging time of 20 h [28]. After filtration, the solids were dried at 80 °C and then calcined at 850 °C. To reduce the powder particle size, the powder was ground for 3 h in a ball mill using a high-density polyethylene (HDPE) milling jar and yttrium-stabilized zirconia grinding media. The macroporous sample models (10.5 mm-diameter round disk and 1.8 mm thick, 500 µm macro pores size) were designed by CAD software (Figure 4a). The models were then sliced (layer thickness = 50 μm) using Creation Workshop software (Datatree3D, Dallas, TX, USA). The data were transferred to the SLA software and equipment (CryoCeram Printer; CryoBeryl Software, Bry, France) for additive manufacturing. The pellets were printed by stereolithography using a high dry matter content slurry (65% dry matter content *w/w*) prepared with the synthetic β-TCP and a photosensitive (λ= 350–400 nm) acrylic resin (CryoBeryl Software, France). The curing energy was set to 5 mW/cm^2^ with a slice thickness of 50 μm. Printing was followed by a series of washing steps in order to remove unpolymerized matter. The pellets were then thermally rebound and sintered at 1050 °C with a heating rate of 5 °C/min at atmospheric air. The printed pellets are illustrated in Figure 4b.

#### 4.2.2. Pellet Characterization

Calcium phosphate ratios (Ca/P) were determined by X-ray diffraction analysis (PanalyticalX’Pert PRO – Copper tube) using the intensity ratio of lines of hydroxyapatite (HA) (the natural mineral form of calcium apatite), (211)/tricalcium phosphate (TCP) (0210) according to the method of the proportioned additions [34,35]. The presence of calcium pyrophosphate (Ca_2_P_2_O_7_) was checked by infrared spectroscopy on a Fourier transform spectrometer (Jasco FT/IR-4600). The absence of calcium oxide traces in the TCP phase was checked by the phenolphthalein test [36].The synthetic β-TCP powder was mainly composed of β-TCP with a low percentage of HA (1.0 ± 0.5%). The macro-porosity of 50% was designed by the 3D model. The samples density was determined by hydrostatic weighing using Archimedes principle under water (3 vacuum cycles, *n* = 5) with a high-precision balance (ALT-310-4 AM; Kern, Germany). The resulting open micro-porosity of the pellet was 24.1 ± 1.0%. Therefore, the total open porosity was 74.1%.

#### 4.2.3. Scanning Electron Microscopy

β-TCP pellets were examined by scanning electron microscopy (SEM) for surface morphologies at 15 kV (JEOL NeoScope JCM 600, Nikon Co., Tokyo, Japan). The pellets were previously air-dried and coated with Ag under vacuum.

#### 4.2.4. Loading of λvir Phages and Alginate Coating of the β-TCP Pellets

**Figure 5:**
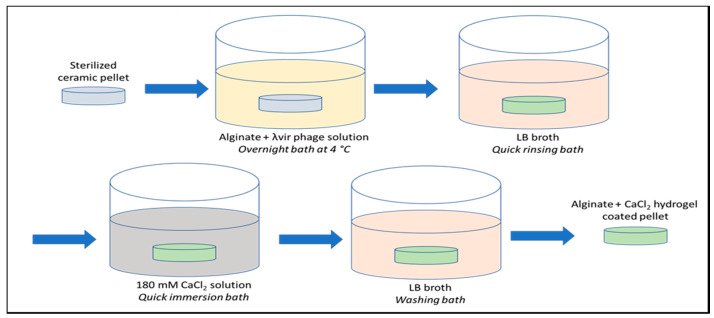
Schematic diagram of alginate–CaCl_2_-hydrogel-coated pellet preparation.

Alginate (Degussa Texturant Systems, France) + λvir phage solutions at 0.5% and 1.0% (*w/v*) alginate concentration were prepared mixing the λvir-phage stock solution with a sterilized aqueous solution of alginate at 2.0% (*w/v*) using the appropriate proportions. The pellets were previously sterilized by immersion in an ethylic alcohol bath (70% (*v/v*)) for 45 min and then air dried. The β-TCP pellets were then bathed in alginate + λvir-phage solutions overnight at 4 °C (Figure 5).

After a quick LB rinsing, the ceramic pellets were then immersed into a 180 mM calcium chloride (VWR ProlaboNormapur, Søborg, Denmark) solution, as the gelling agent for alginate coating. After 3 h of washing bath in LB broth, the pellets were poured into tubes containing 10 mL of LB for the negative control or into a new two-generation culture of *E. coli* A324 at around OD_620nm_ = 0.20 for assessing the lytic activity at 37 °C under 170 rpm shaking. The washing effect in LB broth was evaluated for 0, 3, 6, 18, 24, 48, 72, 144, and 288 h for the control pellets and the 1% alginate–CaCl_2_-coated pellets.

### 4.3. Washing Tests

Instead of the LB broth quick rinse bath, a 3-h wash was also performed to assess the phage release of the loaded ceramic pellets. The analysis of the washed and non-washed pellets was conducted by solid tests on LB dishes for direct enumeration and by kinetic tests on *E. coli* A324 liquid cultures to evaluate the impact of the washing on the lytic activity.

### 4.4. Optical Density Measurements of Bacteria Kinetics

Bacterial growth was measured by optical density (OD) at 620 nm (OD_620_). *E. coli* A324 strain was grown in 10 mL of LB at 37 °C under 170 rpm shaking with a starting OD_620_ close to= 0.02. At around OD_620_ = 0.20 corresponding to a two-generation culture, phage-loaded naked or alginate-coated β-TCP pellets were added into the culture tubes. Growth and lytic kinetics were followed with the spectrophotometer until complete lysis of bacteria or until stationary phase.

### 4.5. Determination of the Phage Titer Load of the Pellets

Bacterial lysis was measured according to the same counting method as for the quantification of the λvir-phage titer. Prior to the enumeration of the phage loading, phages were removed from the alginate–CaCl_2_ covered pellets by 3 successive phosphate buffer washings. The first washing was performed with a stripping buffer (1M NaH_2_PO_4_, 0.15M NaCl, pH = 7) followed by two washings in a desorption buffer (0.2M NaH_2_PO_4_, 0.15 NaCl, pH = 7). The eluates were collected and pooled. The pellets were then put in contact with an *E. coli* liquid culture at 0D_620_ = 0.02 to confirm the absence of any remaining λvir phages. A final elution of the pellets was also performed, and the eluate was controlled by confirming an OD_269_ = 0. The enumeration was then performed according to the same method as described above. Bacterial lysis was also measured by the standard disk-diffusion method [33]. After 24 h of incubation at 37 °C, lysis plaques were counted or measured as the total diameter of the lysis plaque including the sample.

### 4.6. Statistical Analysis

Statistical analysis was performed using OriginPro software 8.5. The data were obtained by at least three replicates and two repetitions (*n* = 3 × 2). The statistical significance of all the data was analyzed with ANOVA. Statistical significance was defined as *p*-value < 0.05.

## 5. Conclusions

This study showed the effectiveness of an alginate coating on phage-loaded bone substitutes to enhance the lytic activity of the phages. We showed that the use of a 1% alginate–CaCl_2_ hydrogel on a bone ceramic material immediately improves the lytic activity and allows a longer releasing of the phages over time. Other combinations of biomaterials and phage-loaded hydrogels, with phage cocktails, or in synergy with antibiotics or other antibacterial molecules, should be investigated in the future. The higher level of lytic activity as well as its prolongation and control could open up promising prospects in preventive and curative therapies.

## Figures and Tables

**Table 1:** Titration of phages retained on β-TCP ceramic pellets after 3 h of lysogenic broth (LB) washing.

Pellets (*n* = 3 × 2)	Phage Concentration (±standard deviation) (PFU/mL)
Control without alginate	1.3 × 10^6^ (±0.2%)
Coated with 0.5% alginate	2.3 × 10^6^ (±0.5%)
Coated with 1.0% alginate	1.3 × 10^7^ (±0.6%)
